# Association of Life's Essential 8 with all-cause mortality and risk of cancer: a prospective cohort study

**DOI:** 10.1186/s12889-024-18879-y

**Published:** 2024-05-27

**Authors:** Jinguo Jiang, Ning Ning, Yang Liu, Zhiwei Cai, Maoxiang Zhao, Xinyi Peng, Liuxin Li, Shuohua Chen, Jing Wang, Feng Wang, Xueying Qin, Yanan Ma, Shouling Wu

**Affiliations:** 1 Department of Clinical Epidemiology, Shengjing Hospital of China Medical University, No. 36, San Hao Street, Heping District, Shenyang, Liaoning Province 110004 China; 2 Department of Epidemiology and Biostatistics, School of Public Health, China Medical University, No.77 Puhe Road, Shenyang North New Area, Shenyang, 110122 Liaoning Province China; 3 Department of Epidemiology and Biostatistics, School of Public Health, Peking University, 38# Xueyuan Road, Haidian District, Beijing, 100191 China; 4 Key Laboratory of Epidemiology of Major Diseases (Peking University), Ministry of Education, Beijing, 100191 China; 5 Department of Cardiology, Second Affiliated Hospital of Shantou University Medical College, Shantou, Guangdong China; 6 Interventional Center of Valvular Heart Disease, Beijing Anzhen Hospital, Capital Medical University, Beijing, China; 7 Hypertension Center, Fuwai Hospital, State Key Laboratory of Cardiovascular Disease of China, National Center for Cardiovascular Diseases of China, Chinese Academy of Medical Sciences and Peking Union Medical College, Beijing, 100037 China; 8 Graduate School, North China University of Science and Technology, Tangshan, China; 9 Department of Cardiology, Kailuan General Hospital, No.57 Xinhua East Road, Tangshan, 063000 Hebei Province China; 10 Peking University Medical Informatics Center, Peking University, Beijing, China; 11 Chinese Center for Health Education, Beijing, China

**Keywords:** Life's essential 8, Longitudinal analysis, Trajectory analysis, Cancer, Death, Threshold effect

## Abstract

**Background:**

No study has concentrated on the association of LE8 with cancer risk and death. We aim to examine the association of LE8 with death and cancer.

**Methods:**

A total of 94733 adults aged 51.42 ± 12.46 years and 77551 participants aged 54.09±12.06 years were enrolled in longitudinal and trajectory analysis respectively. Baseline LE8 was divided into three groups based on the American Heart Association criteria and three trajectory patterns by latent mixture models. We reviewed medical records and clinical examinations to confirm incident cancer during the period from 2006 to 2020. Death information was collected from provincial vital statistics offices. Cox models were used.

**Results:**

12807 all-cause deaths and 5060 cancers were documented during a 14-year follow-up. Relative to participants with high LE8 at baseline, participants with lower levels of LE8 have a significantly increased risk of mortality and incident cancer. All these risks have an increasing trend with LE8 level decreasing. Meanwhile, the trajectory analysis recorded 7483 all-cause deaths and 3037 incident cancers after approximately 10 years. The associations of LE8 with death and cancer were identical to the longitudinal study. In the subtype cancer analysis, LE8 has a strong effect on colorectal cancer risk. Moreover, the cut point is 56.67 in the association between LE8 and death, while the cut point altered to 64.79 in the association between LE8 and incident cancers. These associations were enhanced among younger adults.

**Conclusions:**

There was a significant association of LE8 with death and cancer risk, especially for the young population.

**Supplementary Information:**

The online version contains supplementary material available at 10.1186/s12889-024-18879-y.

## Background

Cancer is one of the biggest killers of human health and the global burden of cancer continues to increase, mainly due to aging, lifestyle, etc [1]. About 4.5 million new cancer cases and 3 million cancer deaths occurred in 2020, ranking among the highest worldwide in China [2]. Cancer prevention remains a significant and challenging public health concern, which may be vital in decreasing death and extending lifespan. Many factors, such as diet [3], physical activity (PA) [4], body mass index (BMI) [5], metabolic factors [5], and smoking [6], are established risk factors for many types of cancer.

In 2010, the American Heart Association (AHA) devised a strategic impact goal to promote cardiovascular health (CVH) in the population and individuals and constructed a measurement of CVH, Life's Simple 7 (LS7), to precisely estimate. To meet this target, the concept of CVH consisted of smoking, PA, BMI, dietary habits, total cholesterol (TC), blood pressure (BP), and fasting blood glucose (FBG) [7]. With the accumulating experience and evidence, LS7 was not enough to represent the full range of CVH. The AHA subsequently included sleep health as another essential component and quantified each metric with a new scoring algorithm ranging from 0 to 100, proposing an updated and enhanced definition of CVH--- now called "Life's Essential 8" (LE8) [8]. Although the health metrics were selected primarily due to their strong associations with CVD, many are associated with cancer and death [9, 10].

No previous study has specifically evaluated the association of LE8 with incident cancer and all-cause mortality. The dose-response effects of LE8 on incident cancer and death are also still unknown, which may help effectively separate the high-risk population who are likely to develop cancer and death in routine medical examination. To meet these challenges, we aim to examine the association of LE8 with cancer incidence and all-cause mortality in a community-based cohort in Kailuan, China.

## Methods

### Study population

The Kailuan study is an ongoing prospective cohort study, designed to find the potential health factor for Chinese in the Kailuan community in Tangshan, Republic of China. Detailed study design and procedures have been described [11, 12]. From June 2006 to October 2007, 101510 adults (81110 men and 20400 women) aged 18-98 were enrolled in the Kailuan community. All participants completed the investigation mainly consisting of three modules (e.g., standardized questionnaire assessment, clinical examination, and laboratory test) and were followed every two years.

**Fig. 1:**
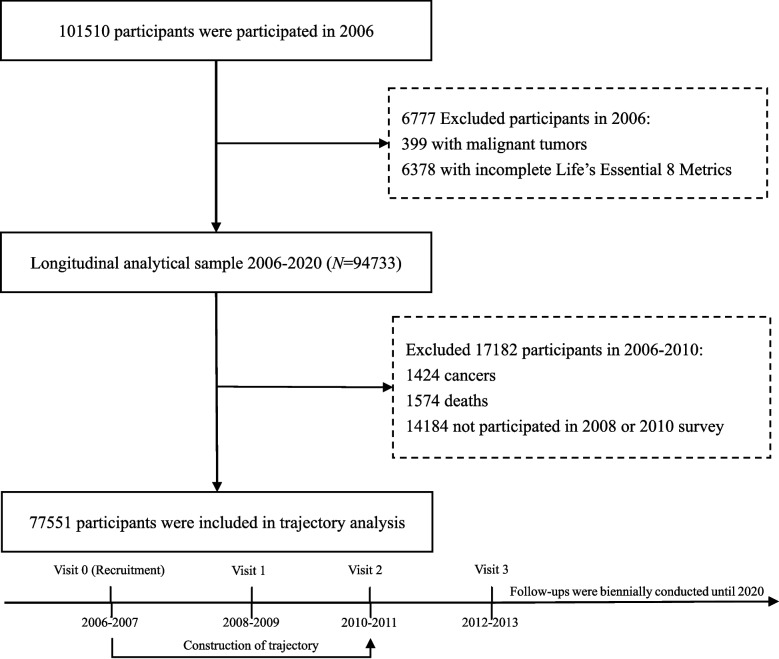
Flow Diagram for Participants Included in the Study

This study evaluated the longitudinal and trajectory association of LE8 with incident cancer and death and further explored the cut-off point to achieve practical value in medical examination. 399 participants suffered from a malignant tumor and 6378 who maintained incomplete LE8 were excluded at baseline. A total of 94733 participants were enrolled in the longitudinal analysis. In addition to the longitudinal study, we further excluded 1424 cancers, 1574 deaths, and 14184 participants with missing data in LE8 from 2006 to 2010 to construct the trajectory pattern. A total of 77551 participants were recruited for trajectory analysis (Fig. 1).

This study complies with the guidelines of the Declaration of Helsinki and the ethics committee of the Kailuan General Hospital approved the study protocol. Participants did not receive financial compensation, and each participant signed informed consent.

### Assessment of Life's Essential 8 and covariates

Trained medical staff collected information on demographic characteristics (sex, date of birth, education level, and occupation), health status (co-morbidity and medication history), lifestyle factors (tobacco consumption, physical activities, sleeping duration, drinking habit, and daily diet), and family history of cancer by face-to-face interview with a standardized questionnaire. We determined the family history of cancer if the response from the interviewee to the question "Did your parents or grandparents have cancer?" was "Yes". Cardiovascular diseases (CVD) included myocardial infarction and stroke; the detailed diagnosis has been published elsewhere [13]. Three education levels were grouped according to the number of years of schooling: ≤ 6, 7-9, and > 9 years, and these corresponded roughly to elementary school or below, middle school, and high school or above, respectively, in this study. The categorization of occupation types was established based on responses to two questions: "Do you work in the mine or not?" and "Are you mainly engaged in manual labor or mental labor?". Participants indicating employment in the mine and engagement in mainly manual labor were classified as coal miners. Those working in the mine but engaged in mainly mental labor were categorized as other blue-collar workers. Participants not working in the mine but mainly involved in manual labor were also classified as other blue-collar workers. Those not working in the mine and primarily engaged in mental labor were categorized as white-collar workers. Participants were inquired about their smoking status: “Do you have the habit of smoking, or have you completely quit?” Further inquiries delved into their smoking frequency over the past year to obtain detailed smoking information according to their response. Drinking status was determined according to participants’ responses to the question, “Did you drink more than 1 time per week in the past half year?”. Dietary habits were assessed through questions regarding flavor preferences, frequency of consuming fatty foods, and frequency of consuming tea. Participants were queried about their weekly frequency of engaging in PA, with the duration of each session being at least 20 minutes. They were also asked to report their daily sleep duration at night in hours. The interviewees were asked whether they had been clinically diagnosed with chronic diseases such as hypertension, diabetes, or other conditions. If the answer was affirmative, further inquiries to obtain detailed information on medication intake. The detailed information regarding the questionnaire survey conducted in the Kailuan study is available elsewhere [11, 14].

Medical workers measured the weight and height of participants and used a uniform formula=weight(kg)/height(m)^2^ to generate the records of BMI. BP was measured according to the seventh Joint National Committee recommendation [15]. After a 5-minute break, BP was measured at least two times with a corrected mercury sphygmomanometer while seated and the average value of BP was used for analysis [16]. Venous blood samples were collected from the antecubital vein after an overnight fast (8-12h). All blood samples were stored at -80°C and the blood biochemistry indices including FBG, TC, and high-density lipoprotein cholesterol (HDL-C) were assessed by the auto-analyzer (Hitachi 747; Hitachi, Tokyo, Japan) at the central laboratory of Kailuan Hospital [12, 17]. Non-HDL-C was calculated using a formula: Non-HDL-C= TC- HDL-C.

Definition and scoring for the components of LE8 which consists of the four health behaviors (daily diet, PA, tobacco exposure, and sleep duration) and four metabolic indicators (BMI, non-HDL, FBG, and BP) had been issued elsewhere [8, 14, 18, 19]. The range of each metric was 0 to 100, and the overall LE8 score was calculated as the unweighted average of all 8 component scores for each participant. Then the participants were grouped by the criteria of the AHA: Low (0-49), Moderate (50-79), and High (80-100) [8]. Moreover, the LE8 trajectory patterns were constructed from 2006 to 2010 using latent mixture models. The confounders were selected based on previous studies [11].

### Assessment of cancer and survival status

The primary outcomes of our study were incident cancer and all-cause death. We obtained the database of cancer diagnoses from the Municipal Social Insurance Institution and Hospital Discharge Register which was updated annually during the follow-up period. Information of histopathological examination, imaging check (e.g., ultrasonography, computerized tomographic scanning, and magnetic resonance imaging), biochemical blood test, and alpha-fetoprotein test were utilized for the cancer diagnosis. The clinical experts were involved in confirming cancer diagnosis by medical records review. All cancers were coded using the International Classification of Diseases, 10th (ICD-10) [20]. The detailed information on cancer cases in the Kailuan study from 2006 to 2020 and the International Classification of Diseases 10th Revision codes has been listed in Table S1. The secondary outcomes were site-specific cancers including lung, liver, gastric, colorectal, and breast cancer. Due to the numbers of other subtypes being limited (n < 200) [21], we cut out the top five and put the rest in another category to avoid the results of limited power. All-cause mortality data was gathered from provincial vital statistics offices and reviewed by physicians [11].

### Statistic analysis

The continuous variables were described by mean ± standard deviation (SD), while frequencies (percentages) were performed for categorized variables. One-way ANOVA and Chi-square tests were conducted to find the difference in each variable as appropriate. The person-time of follow-up for each participant was counted from age at the baseline (2006 for longitudinal study and 2010 for trajectory analysis) until the endpoints [the date of cancer diagnosis, death, or termination of the study (December 31, 2020)] to examine the association between LE8 and cancer risk, while the endpoints were death or termination if we explore the association between LE8 and death.

The statistical processes of primary results consist mainly of three sections: longitudinal analysis to examine the association of baseline LE8 with future cancer and death, trajectory analysis which examines the long-term LE8 patterns and outcomes, and threshold effect analysis to target the cut-off point of LE8 to distinguish the high-risk population.

In the longitudinal analysis, we calculated the incidence density of all-cause death (per 1,000 person-years) and cancer (per 10,000 person-years) across each baseline LE8 status. Cox proportional hazards regression, modeling with age as a time scale since age dramatically impacts the progression of cancer and death [22, 23], was conducted to calculate the hazard ratio (HR) and 95% confidential intervals (CIs) for the association of baseline LE8 with cancer and mortality. Numeric values were assigned to the LE8 level and analyzed as a continuous variable in the adjusted model to examine the trend.

In trajectory analysis, we used a latent mixture model to fit the trajectory pattern of LE8 score from 2006 to 2010 according to previous studies [11, 24, 25]. We checked the assumptions for group-based trajectory modeling and identified three distinct suitable patterns based on previous studies using a trajectory analysis [26].

All Cox proportional hazards models with strata option met Schoenfeld residuals' criteria of proportional hazards assumption before the models were established [27]. We additionally adjusted for potential confounders such as sex, education level, occupation, drinking habit, family history of cancer, and history of CVD.

In the threshold effect analysis, we consider those trajectories flat and unchanged during the exposure period from 2006 to 2010, and therefore, it’s meaningful to use baseline LE8 to target the cut-off points for differentiating high-risk populations who are more likely to develop death and cancer. Firstly, we allocated the population into six groups according to five knots (5th, 25th, 50th, 75th, and 95th centiles) and examined the association of baseline LE8 with incident cancer and death to preliminarily detect the potential dose-response effects. Subsequently, we used restricted cubic splines (RCS) with five knots to flexibly model the association of LE8 with incident cancer and mortality and to describe the dose-response relationships. Furthermore, we used a piecewise function according to five knots to fit each interval and likelihood ratio tests were conducted to determine the non-linearity. As the associations of LE8 with mortality and incident cancer were approximately linear below 50th and 75th, respectively, we additionally leveraged a linear model to calculate HR (95% CI) with per SD of LE8 score increasing.

The robustness of our results has been confirmed in several sensitivity analyses: (1) Stratified analyses were performed for age (<45, 45-65, >65), sex (women, men), year of education (≤6, 7-12, >12 years), occupation (coal miners, other blue collars, white-collar), family history of cancer (no, yes), and history of CVD (no, yes) in the fully adjusted model. In addition, Wald Chi-square tests were conducted to estimate whether covariates modified the association of LE8 with incident cancer and all-cause mortality. (2) All-cause death and incident cancer were simultaneously modeled as the different events in the adjusted Fine-Gray model, which could fully account for the competing risk of all-cause death; (3) We considered the potential for unmeasured confounding between LE8 score and incident cancer or all-cause mortality by calculating E-values [28]. The E-value quantifies the required magnitude of an unmeasured confounder that could negate the observed association between quartiles of LE8 score and incident cancer or all-cause mortality. (4) While previous studies have extensively explored the associations of each component of LE8 with cancer and all-cause death [3–6], it remains beneficial to discuss the association between each factor of LE8 and incident cancer and all-cause mortality. Furthermore, we have explored whether these associations exhibit consistency across different sexes. (5) We employed ROC curve analysis in our study to assess the performance of LE8 as a tool for estimating the risk of mortality or cancer incidence. This analysis also aimed to identify the optimal cut-off point for enhanced diagnostic precision. (6) Since LE8’s trajectory would be insufficient having only three time points, we additionally have extended the period of trajectory construction of LE8 to five years re-fitted the trajectory patterns, and then evaluated the association of LE8 trajectory patterns with incident cancer and all-cause death.

The data were analyzed using Stata/MP2 V17 (Stata Corp LLC, Texas, TX, United States), and the trajectories were modeled using SAS 9.4 (SAS Institute, Cary, NC) with the "Proc Traj procedure", and ROC curve analysis was conducted with MedCalc 19.20. Statistical significance was determined by a two-sided *P*<0.05.

## Results

### Characteristics of the participants

**Fig. 2:**
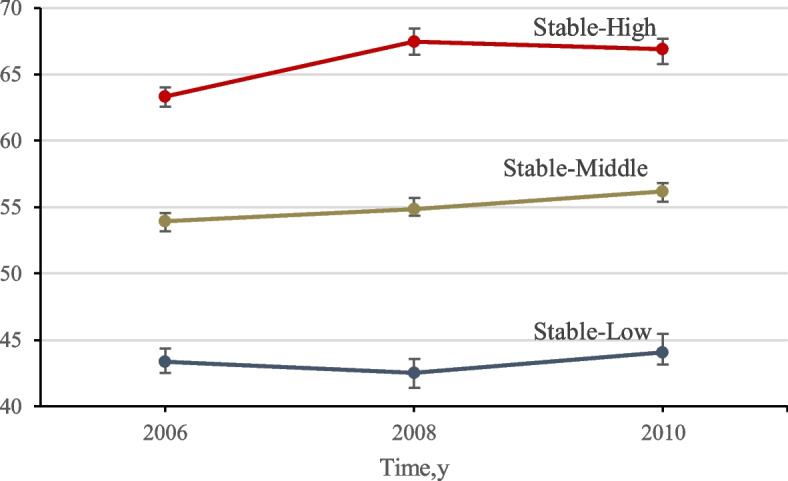
Mean Life’s Essential 8 Score in 2006, 2008, and 2010, According to 3 Life’s Essential 8 Score Trajectory Patterns. The Life’s Essential 8 Score range from 0 to 100, with the highest score representing the best health level. Error bars indicate 95% CI

The average all-cause mortality rate was 10.20 per 1000 person-years, while the average cancer incidence was 40.95 per 10000 person-years. A total of 94733 participants free of cancer, aged 51.43±12.46 years and 79.78% men, were included in the 14-year longitudinal study. The participants with high LE8 were younger, women, educated, white collars, never drinking, and without a family history of cancer (Table 1). In the trajectory analysis, 77551 participants, aged 54.09±12.06 and 78.45% men, met the inclusion such as having completed LE8 data and being free of cancer from 2006 to 2010. The study population was grouped by three discrete trajectories of LE8, and groups were named "Stable-Low," "Stable-Moderate," and "Stable-High," respectively (Fig. 2). The population in "Stable-High" were younger, women, with high social status, white collars, never drinking, and without a family cancer history (Table S2). In addition, the participants who developed cancer from 2006 to 2020 tended to be older (Table S3).

**Table 1:** Basic Characteristics of Participants According to Baseline Life’s Essential 8 Status Introduced by the American Heart Association in the Kailuan cohort

Characteristic	Total	Life’s Essential 8 Status			*P*-value
		Low	Moderate	High	
N	94733(100)	9320(9.84)	75038(79.21)	10375(10.95)	
Age (years)	51.43±12.46	52.28±10.49	52.08±12.31	45.93±13.73	<0.001
Gender, %					<0.001
Women	19155(20.22)	414(4.44)	13658(18.20)	5083(48.99)	
Men	75578(79.78)	8906(95.56)	61380(81.80)	5292(51.01)	
Education level, %					<0.001
Elementary school or below	10251(10.83)	1721(18.49)	8002(10.67)	528(5.09)	
Middle school	65488(69.18)	5988(64.34)	53108(70.82)	6392(61.64)	
High school or above	18928(19.99)	1598(17.17)	13880(18.51)	3450(33.27)	
Occupation, %					<0.001
Coalminers	28890(30.54)	4786(51.39)	22584(30.14)	1520(14.67)	
Other blue collars	58475(61.81)	3961(42.53)	47034(62.78)	7480(72.17)	
White collars	7232(7.65)	567(6.09)	5301(7.08)	1364(13.16)	
Alcohol consumption, %					<0.001
Never	55751(58.88)	2471(26.53)	44960(59.95)	8320(80.20)	
Past	3668(3.87)	662(7.11)	2868(3.82)	138(1.33)	
Current	35264(37.24)	6181(66.36)	27167(36.23)	1916(18.47)	
Family history of cancer, %					<0.001
No	92930(98.11)	9080(97.44)	73675(98.20)	10175(98.07)	
Yes	1789(1.89)	239(2.56)	1350(1.80)	200(1.93)	
History of cardiovascular diseases, %					<0.001
No	91455(96.54)	8665(92.97)	72523(96.65)	10267(98.96)	
Yes	3278(3.46)	655(7.03)	2515(3.35)	108(1.04)	
Components of Life’s Essential 8					
Diet score	38.72±15.20	34.90±17.40	38.76±14.78	41.83±15.33	<0.001
Physical activity score	53.43±24.43	38.55±29.78	54.41±23.41	59.79±21.16	<0.001
Sleep score	87.20±22.26	69.25±29.60	88.16±21.15	96.36±11.01	<0.001
Nicotine exposure score	63.49±45.92	15.51±31.98	64.82±45.37	97.08±15.03	<0.001
Body weight score	67.53±24.68	51.76±21.34	66.43±23.86	89.67±17.64	<0.001
Blood glucose score	85.12±24.38	64.31±29.91	85.88±23.48	98.30±8.39	<0.001
Blood lipids score	73.45±28.63	48.70±27.89	73.66±27.78	94.16±14.94	<0.001
Blood pressure score	45.63±34.22	22.06±24.462	43.35±32.49	83.26±23.85	<0.001
Overall Life’s Essential 8 score	64.32±11.42	43.12±5.01	64.43±7.62	82.56±3.36	<0.001

Values were means ± SD or n (percentages)

### Association of baseline and trajectory LE8 with all-cause mortality

**Fig. 3:**
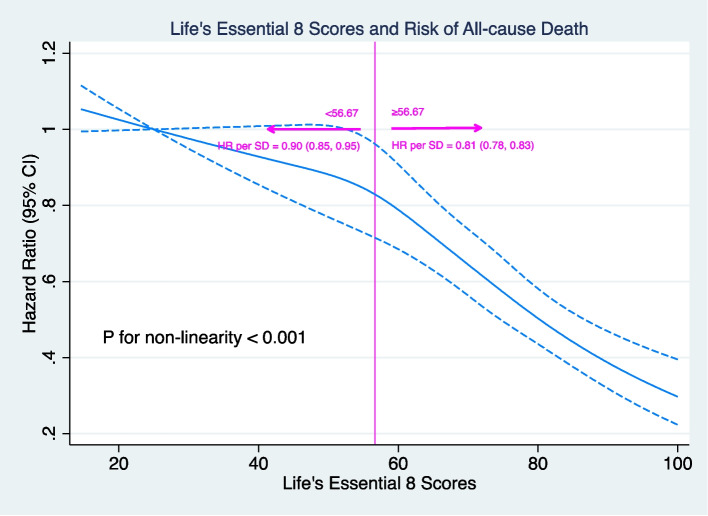
Association between Life’s Essential 8 and All-cause Mortality. Solid blue lines are multivariable adjusted hazard ratios, the dashed blue lines showing 95% confidence intervals derived from restricted spline cubic spline regressions with five knots (5^th^, 25^th^, 50^th^, 75^th^, and 95^th^ percentile). All models were fully adjusted

After a median follow-up of 14.49 years, 1255175.08 person-years and 12807 (13.52%) deaths were observed. In brief, participants who had lower baseline LE8 levels were associated with a higher risk of death and all-cause mortality was increased with LE8 decreasing (*P*-trend <0.001) (Table 2). Relative to the high LE8 level, HRs for the low one were 1.71 (95% CI: 1.56, 1.87) in longitudinal analysis and 1.68 (95% CI: 1.55, 1.81) in trajectory analysis. In the six-group analysis, the risk of death is rising with LE8 declining (*P*-trend <0.001) but the effects of LE8 decreasing on death may increase quicker if the LE8 drops from 100 to 56.67 (25th centile) by comparing the effect values in five groups (Table 2). The RCS further examined and visualized the relation of LE8 with all-cause death, which showed that the HR is decreasing markedly with the LE8 increasing per SD (HR:0.81, 95% CI: 0.78, 0.83) when the LE8 is higher than 56.67 (*P* for non-linearity <0.001) (Fig. 3).

**Table 2:** Hazard Ratios of Life’s Essential 8 with Risk of All-cause Death

Exposure	No. of cases/ Total	Incidence rate per 1000 Person-Years	HR (95% CI)		
			Model I^ab^	Model II^ac^	Model III^ad^
Baseline LE8					
High	749/10375	5.33	1.0 (Reference)	1.0 (Reference)	1.0 (Reference)
Moderate	10408/75038	10.48	1.55 (1.43, 1.67)	1.44 (1.34, 1.56)	1.46 (1.36, 1.58)
Low	1650/9320	13.61	1.83 (1.68, 2.00)	1.62 (1.48, 1.77)	1.71 (1.56, 1.87)
* P-*trend			<0.001	<0.001	<0.001
Fifth of LE8^a^					
6(highest)	304/5201	4.30	1.0 (Reference)	1.0 (Reference)	1.0 (Reference)
5	1479/15471	7.11	1.42 (1.25, 1.61)	1.31 (1.16, 1.48)	1.29 (1.14, 1.46)
4	3591/28528	9.47	1.69 (1.50, 1.90)	1.50 (1.33, 1.69)	1.48 (1.31, 1.66)
3	3344/21455	11.84	2.01 (1.79, 2.27)	1.77 (1.57, 1.99)	1.82 (1.62, 2.05)
2	3237/19400	12.74	2.09 (1.85, 2.35)	1.79 (1.59, 2.02)	1.91 (1.69, 2.15)
1(lowest)	852/4678	14.05	2.24 (1.96, 2.56)	1.86 (1.63, 2.13)	2.03 (1.77, 2.32)
* P-*trend			<0.001	<0.001	<0.001
LE8 Trajectories					
Stable-High	1207/19881	6.25	1.0 (Reference)	1.0 (Reference)	1.0 (Reference)
Stable-Moderate	4510/43718	10.81	1.42 (1.33, 1.51)	1.34 (1.26, 1.43)	1.36 (1.27, 1.45)
Stable-Low	1766/13952	13.41	1.66 (1.54, 1.79)	1.57 (1.46, 1.69)	1.68 (1.55, 1.81)

*Abbreviations*: *LE8*, Life’s Essential 8; *HR* Hazard ratio; *CI* Confidence interval

^a^Fifth means the intervals were determined by five knots (5^th^, 25^th^, 50^th^, 75^th^, and 95^th^ percentile)

^b^Adjust for Age (as time scale) and Gender (women, men)

^c^Adjust for Age (as time scale), Gender (women, men), Education level (elementary school or below, middle school, high school or above), and Occupation (coal miners, other blue collars, white-collar).

^d^Adjust for Age (as time scale), Gender (women, men), Education level (elementary school or below, middle school, high school or above), Occupation (coal miners, other blue collars, white-collar), Drinking (never, abstainer, current), Family history of cancer (No, Yes), and History of cardiovascular diseases (No, Yes)

In the interaction analysis, the associations between LE8 and all-cause death are modified by age, sex, education level, and drinking status (*P*-interaction <0.05). People aged less than 45 years old, women, having high education levels, and non-drinking were more sensitive to the health effect of LE8 on death, which means the association was enhanced among people with those characteristics (Table S4 and Table S5).

### Association of baseline and trajectory LE8 with incident cancer

**Fig. 4:**
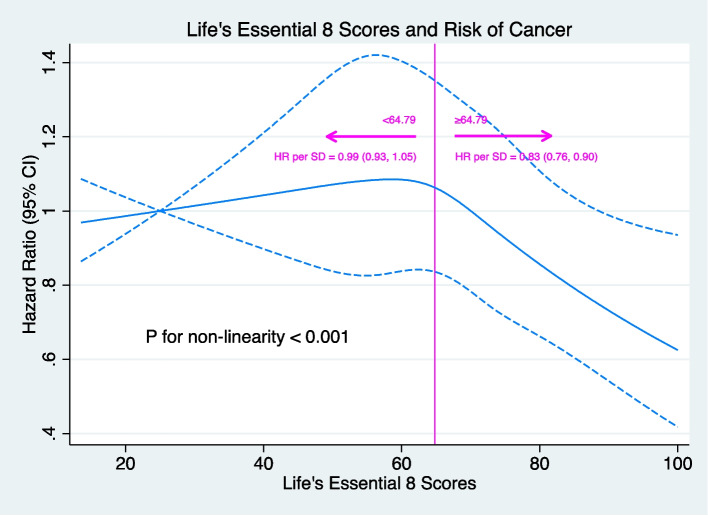
Association between Life’s Essential 8 and Incident Cancer Events. Solid blue lines are multivariable adjusted hazard ratios, the dashed blue lines showing 95% confidence intervals derived from restricted spline cubic spline regressions with five knots (5^th^, 25^th^, 50^th^, 75^th^, and 95^th^ percentile). All models were fully adjusted

After a median follow-up of 14.02 years, 1235548.68 person-years, and 5060 (5.34%) cancer cases were observed, the main results of cancer were as identical as death. The participants who lived with low LE8 were more likely to develop cancer compared with high LE8 participants (Table 3). The incidence of cancer was inversely associated with LE8 level (*P*-trend<0.001) and HRs for the low LE8 were 1.27 (95% CI: 1.11, 1.45) in longitudinal analysis and 1.27 (1.13, 1.43) in trajectory analysis compared with the highest one in the complete models. In the six-group analysis, the same trend was also apparent (*P*-trend<0.001) but the association between baseline LE8 and cancer was not significant (HR:1.04, 95% CI: 0.89, 1.21) when we used the population belonged to 75th to 95th centiles of LE8 compared with the people located in the highest group (Table 3). The RCS further explored this dose-response association to a flexible model, and the graph visualized the relation of LE8 with incident cancer. The HR of LE8 and cancer started to decrease rapidly with per SD of LE8 increased (HR:0.83, 95% CI: 0.76, 0.90) from around 64.79 (50^th^ percentile) to 100 of LE8 (*P* for non-linearity <0.001) (Fig. 4).

**Table 3:** Hazard Ratios of Life’s Essential 8 with Risk of Cancer

Exposure	No. of cases/ Total	Incidence rate per 10,000 Person-Years	HR (95% CI)		
			Model I^ab^	Model II^ac^	Model III^ad^
Baseline LE8					
High	435/10375	31.38	1.0 (Reference)	1.0 (Reference)	1.0 (Reference)
Moderate	4114/75038	42.09	1.32 (1.19, 1.46)	1.26 (1,14, 1.39)	1.26 (1.14, 1.39)
Low	511/9320	42.76	1.33 (1.16, 1.51)	1.26 (1.10, 1.44)	1.27 (1.11, 1.45)
* P-*trend			<0.001	0.001	0.001
Fifth of LE8^a^					
6(highest)	219/5201	31.40	1.0 (Reference)	1.0 (Reference)	1.0 (Reference)
5	711/15471	34.63	1.09 (0.94, 1.27)	1.04 (0.89, 1.21)	1.04 (0.89, 1.21)
4	1576/28528	42.30	1.33 (1.15, 1.53)	1.23 (1.07, 1.43)	1.23 (1.07, 1.42)
3	1193/21455	42.94	1.34 (1.16, 1.56)	1.26 (1.08, 1.46)	1.26 (1.08, 1.46)
2	1109/19400	44.32	1.38 (1.19, 1.61)	1.29 (1.11, 1.49)	1.30 (1.12, 1.51)
1(lowest)	252/4678	42.14	1.31 (1.09, 1.58)	1.22 (1.01, 1.47)	1.24 (1.02, 1.49)
* P-*trend			<0.001	<0.001	<0.001
LE8 Trajectories					
Stable-High	672/19881	35.19	1.0 (Reference)	1.0 (Reference)	1.0 (Reference)
Stable-Moderate	1778/43718	43.14	1.22 (1.11, 1.34)	1.19 (1.08, 1.30)	1.19 (1.08, 1.30)
Stable-Low	587/13952	45.10	1.27 (1.13, 1.43)	1.26 (1.12, 1.42)	1.27 (1.13, 1.43)

*Abbreviations*: *LE8* Life’s Essential 8, *HR* Hazard ratio, *CI* Confidence interval

^a^Fifth means the intervals were determined by five knots (5^th^, 25^th^, 50^th^, 75^th^, and 95^th^ percentile)

^b^Adjust for Age (as time scale) and Gender (women, men)

^c^Adjust for Age (as time scale), Gender (women, men), Education level (elementary school or below, middle school, high school or above), and Occupation (coal miners, other blue collars, white-collar)

^d^Adjust for Age (as time scale), Gender (women, men), Education level (elementary school or below, middle school, high school or above), Occupation (coal miners, other blue collars, white-collar), Drinking (never, abstainer, current), Family history of cancer (No, Yes), and History of cardiovascular diseases (No, Yes)

In the analysis for subtype cancer, we found an interesting result that the baseline LE8 level was inversely associated with cancer in the digestive system, including gastric cancer (GC) (HR:1.83, 95% CI: 1.02, 3.26) and colorectal cancer (CRC) (HR:1.98, 95% CI: 1.16, 3.36), which means LE8 and the progression of digestive cancer may have shared mechanism. Similar results were also found in the trajectory analysis. Still, if participants stood at a low LE8 level for an extended period, the elevated risk of lung cancer would stand out (HR:1.38, 95% CI: 1.10, 1.73) compared to people who maintained a high level of LE8 (Table S6 and Table S7).

In the interaction analysis, we found that the associations between LE8 and incident cancer are modified by age (*P*-interaction <0.05). This association is much stronger among people aged <45 than among other age groups. The same effect was not detected in other variables (Table S8 and Table S9).

### Sensitivity analyses

In the several sensitivity analyses, the robustness was further examined. Stratification analysis further confirmed lower LE8 level was related to a higher risk of cancer and death although part of the results was not significant due to the power of test decline (Table S4, Table S5, Table S8, and Table S9). When considering the competing risk of death in the current study, the results were virtually unchanged in adjusted Fine-Gray models. The sub-distribution hazard ratios of LE8 and cancer are 1.24 (95% CI: 1.08, 1.41) and 1.25 (95% CI: 1.11, 1.40) in longitudinal and trajectory analysis (Table S10). We calculated E-values to estimate the sensitivity to unmeasured confounding. The primary results were robust enough (Table S11). The impacts of eight components on the risk of mortality or cancer incidence varied across different factors (Table S12 and Table S13). LE8 demonstrated a discernment capability, yielding an AUC of 0.577 for death and an AUC of 0.521 for incident cancer, both statistically significant (All *P* value <0.001), although the differences were not very substantial. Additionally, noteworthy was the variation in optimal cut-off points, with LE8 values of 64.17 for all-cause death and 70.00 for incident cancer (Figure S1), indicating differing effects of LE8 on distinct outcomes. Upon extending the trajectory construction period to six years, we identified four distinct trajectories following statistical rules for trajectory construction (Figure S2). Notably, these results remained consistent with our primary findings (Table S14 and Table S15).

## Discussion

### Main findings

This study revealed that LE8 is inversely associated with incident cancer and all-cause mortality. LE8 also has a stronger effect on digestive cancers such as GC and CRC than other cancers, which means the benefit of improvement in LE8, overall CVH, also extends to reinforce the function of the digestive system and the shared pathway may exist between cardiovascular section and digestive process [29]. In addition, there were threshold effects of LE8 on death and incident cancer, and the risk of death and cancer saw a significant rise to a certain degree. This finding can be helpful for clinical workers to distinguish the potential high-risk interviewees as an effective screening tool. Moreover, we observed a strong effect of the LE8 score on the younger individuals and participants with a family history of cancer. As mentioned above, the measurement of LE8 should be given in clinical practice and primary health care, especially for young people, which may hold the key to achieving primary prevention of cancers and extending the human lifespan. The diverse components exerted varying impacts on the risk of all-cause death and cancer.

### Comparison with other studies

In this prospective study, the relationships between LE8 and cancer incidence or all-cause mortality were specifically examined. To our knowledge, most studies The Atherosclerosis Risk In Communities study which included 13,253 participants reported that the number of LS7 components at baseline was inversely associated with cancer incidence [10]. Another Framingham Heart Study and Prevention of Renal and Vascular End-Stage Disease study pooled cohort study found higher LS7 scores were associated with a lower risk of future cancer [30]. Inconsistently, the association between higher baseline CVH and lower cancer-related mortality in the Aerobics Center Longitudinal Study did not reach statistical significance [31]. Those studies did not completely shed light on the health effects of LE8 on cancer risk and mortality. Meanwhile, previous studies have demonstrated that better LS7 status was associated with a lower risk of all-cause mortality [32, 33].

The current finding is quite different from another study that stems from the same cohort [11]. We carefully compared two studies and found that the previous study was based on the imperfect LS7 which fails to distinguish inter-individual differences and comprehensively assess the individual’s CVH. LS7 includes seven factors with a rough scoring system, ranging from 0 to 14, while LE8 additionally included sleep quality and precisely measured the CVH from 0 to 100 to meet the contemporary challenges in public health. There is a significant difference in the algorithm of LS7 and LE8, which means developing a practical screening tool is more possible using the LE8 score. Although the health effects of LS7 have been discussed in previous studies, the potential association of CVH using the LE8 score with incident cancer and all-cause mortality was limited. Current data not only addressed these crucial issues but also shed light on an extension of lifespan. The average all-cause mortality observed in our study aligns with findings reported in a pooled analysis from three cohorts in China, where the all-cause mortality was 1024.76 deaths per 100,000 person-years [34]. Furthermore, data released by the National Cancer Center of China in 2022 indicated a crude incidence and age-standardized incidence for cancer at 341.75 and 201.61 cases per 100,000 person-years, respectively [35]. In our study, the crude incidence of cancer was slightly higher, at 409.53 cases per 100,000 person-years. This discrepancy may be attributed to local features and sex, given that the Kailuan cohort predominantly consisted of miners in Tangshan, a northern Chinese city known for heavy industrial and coal mining activities, contributing to long-term environmental pollution. The close association between environmental factors and cancer in terms of mechanism and epidemiology is well-documented [36]. Additionally, many participants in our study were male and exhibited a higher incidence of common cancers, and the cancer death rate was 1.7 times higher in men than in women (225.97 per 100,000 men vs. 136.79 per 100,000 women) [33]. For the individual metrics, we found that BP is the leading contributor to both cancer and all-cause mortality. The association of achieving ideal BP substantially reducing the risk of cancer and death is not novel [30, 32, 33]. However, inconsistent with the previous finding that PA contributes most to mortality in the US population [37], our study confirmed that BP is the strongest driver for reducing all-cause mortality risks within the LE8 framework among Chinese adults. The difference suggested the relevance of adopting locally tailored strategies in the implementation and utility of LE8 metrics to alleviate the burden of mortality.

The three discrete LE8 trajectories from 2006 to 2010 showed no significant overall change over time. Our findings suggest that individual CVH status as assessed by LE8 might barely change in a long time without intervention, which means the implementation of population-based screening programs and frontline physicians could use a single measurement of LE8 to predict the underlying health status. Furthermore, according to the 2015 China Cancer Statistics [38], through decades of substantial efforts in cancer prevention and control, overall age-standardized cancer incidence and mortality rates in China were stable but still at high levels between 2000 and 2011 [39]. It is hypothesized that an essential reason impeding the decline in cancer incidence and mortality might be that some CVD risk factors have not improved significantly.

### LE8 with all-cause mortality

The protective effect of ideal CVH on death may be due partly to attenuating the effects of genetic susceptibility to CVD. A recent study from UK Biobank, based on mediation analyses, elucidated that ideal CVH is associated with lower cardiovascular outcomes and all-cause mortality, and that a more pronounced correlation was observed in individuals with higher genetic susceptibility to CVD [40]. Undoubtedly, this emphasized the importance of maintaining an ideal CVH to reduce the risk of genetic susceptibility to health, especially for those with high genetic risk.

Based on the recently updated LE8, a prospective cohort study, also from Kailuan, showed that baseline and long-term CVH were associated with an inverse gradient in the risk of all-cause mortality in young people under 40 years of age [19]. We detected an association between LE8 and death more significantly in participants <45 years than in those ≥45 years. LE8 was associated with an enhanced benefit of lower all-cause mortality in younger adults, effect sizes were enlarged among the younger group, and the age difference in effects was kept in line with previous studies [41]. In brief, the findings highlight that improving overall CVH status in younger adults could effectively mitigate cardiovascular risk and prolong the life span.

### LE8 with incident cancer

Better CVH at baseline and long-term trajectory are inversely associated with lower cancer risk in the Chinese population. Our study provides additional references for clinicians to guide the prevention of high-risk cancer populations. To our knowledge, there are few age-specific findings on CVH and cancer incidence, but in our study, we observed an association between LE8 and the risk of cancer more significantly in younger adults. This underscores the importance of maintaining healthy behaviors and factors early in life, and improving overall cardiovascular health in young adults can significantly prevent the triggered factors of cancer.

We found an inverse relationship between LE8 and incident cancer, which means there may be some shared mechanisms between CVH and cancer. Increasing evidence supports the role of clonal hematopoiesis [42, 43], inflammatory factor [44], and hyperinsulinemia [45, 46] in the development of CVD and cancer. Clonal hematopoiesis is causally associated with an increased risk of cancer and all-cause mortality, and loss of function in hematopoietic cells and mutations in macrophages accelerate CVD [43]; Interleukin-1β mediates inflammation in the tumor microenvironment and stimulates downstream interleukin-6 receptor signaling pathway to develop CVD [44, 47]; Vascular endothelial growth factor is associated with abnormal endothelial function, angiogenesis, and hemodynamic effects and is often highly expressed in cancer and CVD [45].

Our study further explored the association between LE8 and cancer subtypes and found that LE8 had a stronger effect on digestive cancers (such as GC and CRC) than other cancers. Digestive cancers play a leading role in cancer deaths [48] and there may be some shared underlying pathogenic mechanisms between CVH and the digestive system, and these include common lifestyle-related risk factors such as smoking, obesity, hypertension, and dyslipidemia, which might lead to oxidative stress and chronic inflammation, increasing the risk of both outcomes [49–51]. Shared cellular, signaling, and genetic pathways, such as diet factors might significantly affect the risk of CRC through inflammatory pathways or the intestinal microbial environment [52, 53]; Long non-coding RNAs (lncRNAs) function in different gene regulatory pathways through epigenetic modifications as well as transcriptional and post-transcriptional regulation and their function and expression levels have been proven to be associated with CVD and cancer [54], especially in GC metastasis [55].

### Threshold effects of LE8 on all-cause mortality and cancer risk

Conventional evidence supports a negative dose-dependent association between single baseline CVH and all-cause mortality, suggesting that lower CVH is associated with a higher risk of all-cause mortality compared with higher CVH [56]. However, positive dose-response associations within certain intervals have also been reported [57], which may be related to confounding by unmeasured disease or subclinical symptoms, but regrettably, these LS7 studies have found no threshold effect. Sleep duration has been found to have an inverse L-shaped association with the risk of all-cause mortality, with a threshold effect showing an optimal critical value of approximately 9 h [58]. Our study extended previous investigations by adding sleep indicators and found the optimal threshold based on a negative dose-response association between overall CVH score and risk of cancer and death. The risks of cancer and all-cause mortality were dramatically reduced when baseline and trajectory LE8 scores were above optimal thresholds after controlling for confounders. Our findings suggest that the presence of a critical score quantification point can make a slight improvement in the total CVH score and lead to a significant reduction in cancer and death. This is a remarkably substantial finding in this study. Especially for those who are susceptible to cancer or other chronic diseases, some of them may be unable to obtain consistently high CVH scores due to functional or other limitations, so if they can work toward a threshold score, it could significantly reduce their risk of cancer and death. Thus, the finding of a threshold node for CVH score benefits both cancer and mortality risk improvement, while a lower threshold score for death compared to cancer was observed. That implies that improvements in CVH would be more likely to benefit mortality, a small improvement in total CVH score could lead to a large reduction in mortality. Overall, targeted programs and health policies targeting LE8 factors with lower scores are encouraged to improve the total population-level LE8 score. When LE8 threshold scores are adopted and enhanced by the population, cancer and death risks can be effectively reduced. The discovery of threshold effects improves the sensitivity of measuring CVH changes over time at the individual and population levels and may help researchers, policymakers and health systems develop standardized tools for targeting precision interventions.

## Strengths and limitations

Apart from this study based on a large-scale prospective community-based cohort with a long follow-up period, LE8 was repeatedly collected. This may reduce random errors and shed light on the potential association between long-term LE8 and cancer incidence or longevity. Furthermore, this study is the first to disclose LE8 associated with cancer incidence and mortality. However, the study is limited in several ways. First, all participants were enrolled from the Kailuan Group, and most of them were men, which means the generalizability is limited. Second, the period of trajectory construction was around five years; thus, the life course changes in LE8 could not be observed, which is worth to be depicted in future studies. Third, many confounding factors still have not been controlled in our study. We calculated the E-value via the Stata module for unmeasured confounding to quantify the potential influence of unmeasured confounders in sensitivity analysis. We found that an unmeasured confounder might not account for the entirety of the LE8 effect. Fourth, our present study lacks information regarding the association between LE8 and cause-specific mortality, such as CVD mortality or cancer mortality. This limitation arises from the Kailuan study's failure to collect specific causes of death. Understanding the specific types of mortality associated with LE8 would provide valuable insights. Consequently, future research endeavors should seek more population-based evidence to thoroughly investigate the association between LE8 and cause-specific mortality. Fifth, the limited trajectory period of four years in our study although we extended it to six years, representing only a small fraction of the human lifespan, presented a constraint in fully capturing the comprehensive patterns of LE8 changes across an individual's life. This underscores the significance of exploring the lifetime trajectory of LE8 in future studies to gain a more thorough understanding.

## Conclusions

LE8 was inversely associated with incident cancer and all-cause mortality, especially for younger adults. There are threshold effects of LE8 on cancer and all-cause mortality. The findings augment important information that participants were encouraged to measure LE8 in medical examination, which may help assess their health status; thus, the participants can acquire appropriate treatment promptly, especially for the high-risk population.

### Supplementary Information

Supplementary Material 1. 

## Data Availability

The datasets used and analyzed during the current study are available from the corresponding author on reasonable request.

## Abbreviations

AHA: The American Heart Association
ARIC: Atherosclerosis Risk In Communities
BMI: Body mass index
BP: Blood pressure
CVH: Cardiovascular health
CIs: Confidential intervals
CVD: Cardiovascular disease
FBG: Fasting blood glucose
HR: Hazard ratio
HDL-C: High-density lipoprotein cholesterol
IQR: Interquartile range
LS7: Life's Simple 7
LE8: Life's Essential 8
NHANES: National Health and Nutrition Examination Survey
PA: Physical activity
PH: Proportional hazard
PAFs: Population attributable fractions
SD: Standard deviation
TC: Total Cholesterol
TG: Triglyceride
