# Supplementary material for: Association of Life's Essential 8 with all-cause mortality and risk of cancer: a prospective cohort study
Source: BMC Public Health. 2024 May 27;24:1406. doi: 10.1186/s12889-024-18879-y (PMC11129373; doi:10.1186/s12889-024-18879-y)
Supplement: Supplementary file 1 — Supplementary Material 1. [file 12889_2024_18879_MOESM1_ESM.docx]

**Table S1.** The International Classification of Diseases (10th Version) and Distribution of Subtype Cancers in the Kailuan Study from 2006 to 2020

**Table S2.** Basic Characteristics of Participants by Life’s Essential 8 Trajectories in Kailuan Study

**Table S3.** Basic Characteristics of Participants by Cancer Cases Appeared during Follow-up

**Table S4.** Stratified Analyses of between Baseline Life’s Essential 8 Status and All-cause Death in Full Models**^†^**

**Table S5.** Stratified Analyses of between Life’s Essential 8 Trajectories and All-cause Death in Full Models**^†^**

**Table S6.** Baseline Life’s Essential 8 Status with Risk of Subtypes Cancer

**Table S7.** Life’s Essential 8 Trajectories with Risk of Subtypes Cancer

**Table S8.** Stratified Analyses of between Baseline Life’s Essential 8 Status and Cancer in Full Models**^†^**

**Table S9.** Stratified Analyses of between Life’s Essential 8 Trajectories and Cancer in Full Models**^†^**

**Table S10.** Hazard Ratios of Life’s Essential 8 with Risk of Cancer Using Fine-Gray Models

**Table S11.** The E-value for Life’s Essential 8 and Risk of Cancer or All-cause Death

**Table S12.** Hazard Ratios of Each Component of Life’s Essential 8 with Risk of All-cause Death Stratified by Sex

**Table S13.** Hazard Ratios of Each Component of Life’s Essential 8 with Risk of Cancer Stratified by Sex

**Table S14.** Hazard Ratios of Life’s Essential 8 with Risk of All-cause Death after Extending the Period of Trajectory Construction to Six Years

**Table S15.** Hazard Ratios of Life’s Essential 8 with Risk of Cancer after Extending the Period of Trajectory Construction to Six Years

**Figure S1.** ROC Curves for the Association of Life’s Essential 8 with all-cause Death (right) and Incident Cancer (left)

**Figure S2.** Mean Life’s Essential 8 Score in 2006, 2008, 2010, and 2012, According to 4 Life’s Essential 8 Score Trajectory Patterns

**Table S1.** The International Classification of Diseases (10th Version) and Distribution of Subtype Cancers in the Kailuan Study from 2006 to 2020

| **Types of Cancer (ICD-10)** | **The number (percentage) of cases^†^** |
| --- | --- |
| C00 | 6 (0.12) |
| C01 | 5 (0.10) |
| C02 | 8 (0.16) |
| C03 | 7 (0.14) |
| C04 | 8 (0.16) |
| C05 | 6 (0.12) |
| C06 | 3 (0.06) |
| C07 | 8 (0.16) |
| C08 | 3 (0.06) |
| C09 | 3 (0.06) |
| C10 | 14 (0.28) |
| C11 | 29 (0.57) |
| C12 | 1 (0.02) |
| C13 | 19 (0.38) |
| C14 | 7 (0.14) |
| C15 | 179 (3.54) |
| C16 | 364 (7.19) |
| C17 | 34 (0.67) |
| C18 | 284 (5.61) |
| C19 | 10 (0.20) |
| C20 | 352 (6.96) |
| C21 | 3 (0.06) |
| C22 | 427 (8.44) |
| C23 | 22 (0.43) |
| C24 | 30 (0.59) |
| C25 | 129 (2.55) |
| C30 | 4 (0.08) |
| C31 | 4 (0.08) |
| C32 | 73 (1.44) |
| C34 | 1370 (27.08) |
| C37 | 9 (0.18) |
| C38 | 12 (0.24) |
| C39 | 1 (0.02) |
| C40 | 11 (0.22) |
| C41 | 17 (0.34) |
| C42 | 58 (1.15) |
| C43 | 4 (0.08) |
| C44 | 39 (0.77) |
| C45 | 4 (0.08) |
| C47 | 4 (0.08) |
| C48 | 5 (0.10) |
| C49 | 17 (0.34) |
| C50 | 304 (6.01) |
| C51 | 4 (0.08) |
| C52 | 1 (0.02) |
| C53 | 40 (0.79) |
| C54 | 57 (1.13) |
| C55 | 2 (0.04) |
| C56 | 33 (0.65) |
| C57 | 2 (0.04) |
| C60 | 10 (0.20) |
| C61 | 138 (2.73) |
| C62 | 6 (0.12) |
| C63 | 1 (0.02) |
| C64 | 178 (3.52) |
| C65 | 12 (0.24) |
| C66 | 16 (0.32) |
| C67 | 172 (3.40) |
| C68 | 3 (0.06) |
| C70 | 36 (0.71) |
| C71 | 58 (1.15) |
| C72 | 5 (0.10) |
| C73 | 114 (2.25) |
| C74 | 5 (0.10) |
| C75 | 29 (0.57) |
| C76 | 16 (0.32) |
| C77 | 26 (0.51) |
| C79 | 1 (0.02) |
| C80 | 40 (0.79) |
| C81 | 6 (0.12) |
| C83 | 17 (0.34) |
| C84 | 1 (0.02) |
| C85 | 53 (1.05) |
| C90 | 22 (0.43) |
| C91 | 9 (0.18) |
| C92 | 27 (0.53) |
| C93 | 4 (0.08) |
| C94 | 1 (0.02) |
| C95 | 7 (0.14) |
| D32 | 1 (0.02) |
| D35 | 1 (0.02) |
| D43 | 2 (0.04) |
| D46 | 4 (0.08) |
| D47 | 3 (0.06) |
| **C00-C95, D32-D47** | **5060 (100)** |

Values were n (percentages).

**^†^**The number of cancer cases was counted on participants who had cancer from 2006 to 2020.

**Table S2.** Basic Characteristics of Participants by Life’s Essential 8 Trajectories in Kailuan Study

| Characteristics | Total | Life’s Essential 8 Trajectories | | |  |
| --- | --- | --- | --- | --- | --- |
|  |  | Stable-Low | Stable-Moderate | Stable-High | *P*-value |
| N | 77551(100) | 13952(17.99) | 43718(56.37) | 19881(25.64) |  |
| Age (years) | 54.09±12.06 | 54.61±10.30 | 55.27±11.84 | 51.13±13.15 | <0.001 |
| Gender, % |  |  |  |  | <0.001 |
| Women | 16715(21.55) | 918(6.58) | 6985(15.98) | 8812(44.32) |  |
| Men | 60836(78.45) | 13034(93.42) | 36733(84.02) | 11069(55.68) |  |
| Education level, % |  |  |  |  | <0.001 |
| Elementary school or below | 7183(9.27) | 1743(12.50) | 4314(9.87) | 1126(5.67) |  |
| Middle school | 53837(69.46) | 9661(69.30) | 31282(71.60) | 12894(64.88) |  |
| High school or above | 16485(21.27) | 2536(18.19) | 8095(18.53) | 5854(29.46) |  |
| Occupation, % |  |  |  |  | <0.001 |
| Coalminers | 23685(30.58) | 6321(45.35) | 13850(31.73) | 3514(17.70) |  |
| Other blue collars | 47566(61.42) | 6757(48.48) | 26787(61.36) | 14022(70.62) |  |
| White collars | 6195(8.00) | 860(6.17) | 3015(6.91) | 2320(11.68) |  |
| Alcohol consumption, % |  |  |  |  | <0.001 |
| Never | 44901(57.93) | 4758(34.13) | 25291(57.88) | 14852(74.72) |  |
| Past | 2710(3.50) | 723(5.19) | 1592(3.64) | 395(1.99) |  |
| Current | 29901(38.58) | 8461(60.69) | 16811(38.47) | 4629(23.29) |  |
| Family history of cancer, % |  |  |  |  | <0.001 |
| No | 76028(98.05) | 13593(97.47) | 42932(98.22) | 19503(98.10) |  |
| Yes | 1510(1.95) | 353(2.53) | 780(1.78) | 377(1.90) |  |
| History of cardiovascular diseases, % |  |  |  |  | <0.001 |
| No | 75342(97.15) | 13332(95.56) | 42387(96.96) | 19623(98.70) |  |
| Yes | 2209(2.85) | 620(4.44) | 1331(3.04) | 258(1.30) |  |
| Components of Life’s Essential 8 |  |  |  |  |  |
| Diet score | 38.86±15.28 | 37.09±16.66 | 38.74±14.96 | 40.35±14.82 | <0.001 |
| Physical activity score | 53.13±24.53 | 45.86±27.72 | 53.76±24.05 | 56.84±22.02 | <0.001 |
| Sleep score | 87.53±21.85 | 78.51±26.97 | 88.04±21.26 | 92.73±16.53 | <0.001 |
| Nicotine exposure score | 63.28±46.00 | 28.89±42.63 | 62.65±45.90 | 88.80±29.43 | <0.001 |
| Body weight score | 67.31±24.58 | 52.53±21.01 | 65.13±23.35 | 82.46±21.31 | <0.001 |
| Blood glucose score | 85.65±23.89 | 70.70±29.87 | 86.18±22.89 | 95.00±14.21 | <0.001 |
| Blood lipids score | 73.44±28.58 | 55.40±29.18 | 73.47±27.79 | 86.01±22.46 | <0.001 |
| Blood pressure score | 46.83±34.15 | 28.63±28.26 | 42.78±32.31 | 68.53±30.87 | <0.001 |
| Overall Life’s Essential 8 score | 64.50±11.43 | 49.70±8.05 | 63.84±7.56 | 76.34±6.58 | <0.001 |

Values were means ± SD or n (percentages).

**Table S3.** Basic Characteristics of Participants by Cancer Cases Appeared during Follow-up

| Characteristics | Total | Cancer Status | |  |
| --- | --- | --- | --- | --- |
|  |  | No | Yes | *P*-value |
| N | 94733 (100) | 89673(94.66) | 5060(5.34) |  |
| Age (years) | 51.43±12.46 | 51.12±12.50 | 56.80±10.33 | <0.001 |
| Gender, % |  |  |  | 0.019 |
| Women | 19155(20.22) | 18197(20.29) | 958(18.93) |  |
| Men | 75578(79.78) | 71.476(79.71) | 4102(81.07) |  |
| Education level, % |  |  |  | <0.001 |
| Elementary school or below | 10251(10.83) | 9519(10.62) | 732(14.47) |  |
| Middle school | 65488(69.18) | 61917(69.10) | 3571(70.61) |  |
| High school or above | 18928(19.99) | 18174(20.28) | 754(14.91) |  |
| Occupation, % |  |  |  | 0.047 |
| Coalminers | 28890(30.54) | 27412(30.61) | 1478(29.26) |  |
| Other blue collars | 58475(61.81) | 55269(61.72) | 3206(63.46) |  |
| White collars | 7232(7.65) | 6864(7.67) | 368(7.28) |  |
| Alcohol consumption, % |  |  |  | <0.001 |
| Never | 55751(58.88) | 52721(58.82) | 3030(59.92) |  |
| Past | 3668(3.87) | 3409(3.80) | 259(5.12) |  |
| Current | 35264(37.24) | 33496(37.37) | 1768(34.96) |  |
| Family history of cancer, % |  |  |  | 0.101 |
| No | 92930(98.11) | 87981(98.13) | 4949(97.81) |  |
| Yes | 1789(1.89) | 1678(1.87) | 111(2.19) |  |
| History of cardiovascular diseases, % |  |  |  | 0.003 |
| No | 91455(96.54) | 86608(96.58) | 4847(95.79) |  |
| Yes | 3278(3.46) | 3065(3.42) | 213(4.21) |  |
| Components of Life’s Essential 8 |  |  |  |  |
| Diet score | 38.72±15.20 | 38.72±15.18 | 38.70±15.55 | 0.016 |
| Physical activity score | 53.43±24.43 | 53.27±24.37 | 56.28±25.37 | <0.001 |
| Sleep score | 87.20±22.26 | 87.25±22.21 | 86.30±23.03 | <0.001 |
| Nicotine exposure score | 63.49±45.92 | 63.65±45.86 | 60.67±46.81 | 0.045 |
| Body weight score | 67.53±24.68 | 67.50±24.67 | 68.07±24.83 | 0.526 |
| Blood glucose score | 85.12±24.38 | 85.18±24.36 | 84.05±24.78 | 0.091 |
| Blood lipids score | 73.45±28.63 | 73.45±28.63 | 73.53±28.68 | 0.845 |
| Blood pressure score | 45.63±34.22 | 45.88±34.27 | 41.12±33.03 | <0.001 |
| Overall Life’s Essential 8 score | 64.32±11.42 | 64.36±11.44 | 63.59±11.04 | 0.001 |
| Baseline Life’s Essential 8 status, % |  |  |  | <0.001 |
| High | 10375(10.95) | 9940(11.08) | 435(8.60) |  |
| Moderate | 75038(79.21) | 70924(79.09) | 4114(81.30) |  |
| Low | 9320(9.84) | 8809(9.82) | 511(10.10) |  |

Values were means ± SD or n (percentages).**Table S4.** Stratified Analyses of between Baseline Life’s Essential 8 Status and All-cause Death in Full Models**^†^**

| Subgroups | Life’s Essential 8 status | | | *P*-trend | *P*-interaction |
| --- | --- | --- | --- | --- | --- |
|  | High | Moderate | Low |  |  |
| Age (years) |  |  |  |  | <0.001 |
| <45 | 1.0 (Reference) | 1.92 (1.44, 2.56) | 3.18 (2.25, 4.50) | <0.001 |  |
| 45-65 | 1.0 (Reference) | 1.43 (1.26, 1.61) | 1.96 (1.71, 2.26) | <0.001 |  |
| >65 | 1.0 (Reference) | 1.08 (0.98, 1.20) | 1.29 (1.13, 1.46) | <0.001 |  |
| Gender |  |  |  |  | <0.001 |
| Female | 1.0 (Reference) | 3.13 (2.53, 3.86) | 7.44 (5.48, 10.11) | <0.001 |  |
| Male | 1.0 (Reference) | 1.24 (1.14, 1.34) | 1.42 (1.29, 1.56) | <0.001 |  |
| Education level |  |  |  |  | <0.001 |
| Elementary school or below | 1.0 (Reference) | 1.01 (0.85, 1.18) | 0.96 (0.80, 1.15) | 0.387 |  |
| Middle school | 1.0 (Reference) | 1.42 (1.29, 1.55) | 1.83 (1.64, 2.05) | <0.001 |  |
| High school or above | 1.0 (Reference) | 2.63 (2.10, 3.30) | 3.60 (2.75, 4.71) | <0.001 |  |
| Occupation |  |  |  |  | 0.455 |
| Coalminers | 1.0 (Reference) | 1.16 (0.99, 1.35) | 1.28 (1.09, 1.51) | 0.001 |  |
| Other blue collars | 1.0 (Reference) | 1.51 (1.38, 1.65) | 1.89 (1.69, 2.13) | <0.001 |  |
| White collars | 1.0 (Reference) | 2.08 (1.57, 2.75) | 2.33 (1.64, 3.31) | <0.001 |  |
| Alcohol consumption |  |  |  |  | 0.135 |
| Never | 1.0 (Reference) | 1.57 (1.45, 1.71) | 1.93 (1.72, 2.17) | <0.001 |  |
| Abstainer | 1.0 (Reference) | 1.21 (0.80, 1.84) | 1.39 (0.90, 2.14) | 0.044 |  |
| Current | 1.0 (Reference) | 1.04 (0.88, 1.24) | 1.18 (0.99, 1.42) | 0.001 |  |
| Family history of cancer |  |  |  |  | 0.804 |
| No | 1.0 (Reference) | 1.46 (1.35, 1.57) | 1.71 (1.56, 1.87) | <0.001 |  |
| Yes | 1.0 (Reference) | 1.22 (0.60, 2.48) | 1.72 (0.79, 3.75) | 0.075 |  |
| History of cardiovascular diseases |  |  |  |  | 0.587 |
| No | 1.0 (Reference) | 1.46 (1.35, 1.58) | 1.73 (1.57, 1.90) | <0.001 |  |
| Yes | 1.0 (Reference) | 1.09 (0.79, 1.50) | 1.22 (0.87, 1.70) | 0.073 |  |

**^†^**Adjust for Age (as time scale), Gender (women, men), Education level (elementary school or below, middle school, high school or above), Occupation (coal miners, other blue collars, white-collar), Drinking (never, abstainer, current), Family history of cancer (No, Yes), and History of cardiovascular diseases (No, Yes). The strata option has been implemented to tackle the variable violated the proportional assumption. Of note, the examined variable did not adjust.

**Table S5.** Stratified Analyses of between Life’s Essential 8 Trajectories and All-cause Death in Full Models**^†^**

| Subgroups | Life’s Essential 8 Trajectories | | | *P*-interaction |
| --- | --- | --- | --- | --- |
|  | Stable-Low | Stable-Middle | Stable-High |  |
| Age (years) |  |  |  | <0.001 |
| <45 | 1.0 (Reference) | 1.36 (0.94, 1.97) | 2.67 (1.75, 4.07) |  |
| 45-65 | 1.0 (Reference) | 1.47 (1.31, 1.65) | 2.23 (1.97, 2.53) |  |
| >65 | 1.0 (Reference) | 1.18 (1.09, 1.28) | 1.43 (1.30, 1.59) |  |
| Gender |  |  |  | <0.001 |
| Female | 1.0 (Reference) | 2.96 (2.47, 3.55) | 6.64 (5.24, 8.43) |  |
| Male | 1.0 (Reference) | 1.18 (1.10, 1.26) | 1.41 (1.30, 1.53) |  |
| Education level |  |  |  | 0.002 |
| Elementary school or below | 1.0 (Reference) | 1.01 (0.88, 1.16) | 1.02 (0.87, 1.20) |  |
| Middle school | 1.0 (Reference) | 1.36 (1.25, 1.47) | 1.78 (1.62, 1.96) |  |
| High school or above | 1.0 (Reference) | 2.09 (1.74, 2.52) | 2.85 (2.29, 3.54) |  |
| Occupation |  |  |  | 0.327 |
| Coalminers | 1.0 (Reference) | 1.06 (0.93, 1.19) | 1.27 (1.11, 1.46) |  |
| Other blue collars | 1.0 (Reference) | 1.45 (1.34, 1.58) | 1.86 (1.68, 2.05) |  |
| White collars | 1.0 (Reference) | 1.74 (1.39, 2.18) | 2.00 (1.51, 2.65) |  |
| Alcohol consumption |  |  |  | 0.041 |
| Never | 1.0 (Reference) | 1.48 (1.37, 1.60) | 1.95 (1.76, 2.15) |  |
| Abstainer | 1.0 (Reference) | 0.97 (0.74, 1.27) | 1.07 (0.80, 1.44) |  |
| Current | 1.0 (Reference) | 1.10 (0.96, 1.25) | 1.31 (1.14, 1.50) |  |
| Family history of cancer |  |  |  | 0.616 |
| No | 1.0 (Reference) | 1.35 (1.27, 1.44) | 1.68 (1.55, 1.81) |  |
| Yes | 1.0 (Reference) | 1.79 (0.93, 3.45) | 1.73 (0.84, 3.57) |  |
| History of cardiovascular diseases |  |  |  | 0.187 |
| No | 1.0 (Reference) | 1.36 (1.28, 1.46) | 1.71 (1.57, 1.85) |  |
| Yes | 1.0 (Reference) | 1.10 (0.86, 1.41) | 1.24 (0.95, 1.61) |  |

**^†^**Adjust for Age (as time scale), Gender (women, men), Education level (elementary school or below, middle school, high school or above), Occupation (coal miners, other blue collars, white-collar), Drinking (never, abstainer, current), Family history of cancer (No, Yes), and History of cardiovascular diseases (No, Yes). The strata option has been implemented to tackle the variable violated the proportional assumption. Of note, the examined variable did not adjust.

**Table S6.** Baseline Life’s Essential 8 Status with Risk of Subtypes Cancer

| Subtypes of Cancer | Life’s Essential 8 Status | | |  | |  |
| --- | --- | --- | --- | --- | --- | --- |
|  | High | Moderate | Low | | *P*-trend | |
| Lung cancer |  |  |  | |  | |
| No. of cases/Total | 113/10375 | 1104/75038 | 153/9320 | |  | |
| Incidence rate/10,000 Person-Years | 8.07 | 11.15 | 12.66 | |  | |
| HR (95%CI) **^†^** | 1.0 (Reference) | 1.06 (0.87, 1.29) | 1.09 (0.84, 1.41) | | 0.524 | |
| Liver cancer |  |  |  | |  | |
| No. of cases/Total | 42/10375 | 339/75038 | 46/9320 | |  | |
| Incidence rate/10,000 Person-Years | 2.99 | 3.42 | 3.80 | |  | |
| HR (95%CI) **^†^** | 1.0 (Reference) | 0.79 (0.57, 1.10) | 0.78 (0.50, 1.21) | | 0.306 | |
| Gastric cancer |  |  |  | |  | |
| No. of cases/Total | 19/10375 | 307/75038 | 38/9320 | |  | |
| Incidence rate/10,000 Person-Years | 1.35 | 3.09 | 3.14 | |  | |
| HR (95%CI) **^†^** | 1.0 (Reference) | 1.89 (1.16, 3.05) | 1.83 (1.02, 3.26) | | 0.086 | |
| Colorectal cancer |  |  |  | |  | |
| No. of cases/Total | 22/10375 | 283/75038 | 47/9320 | |  | |
| Incidence rate/10,000 Person-Years | 1.57 | 2.85 | 3.88 | |  | |
| HR (95%CI) **^†^** | 1.0 (Reference) | 1.55 (0.99, 2.42) | 1.98 (1.16, 3.36) | | 0.012 | |
| Breast cancer |  |  |  | |  | |
| No. of cases/Total | 65/10375 | 229/75038 | 10/9320 | |  | |
| Incidence rate/10,000 Person-Years | 4.64 | 2.31 | 0.83 | |  | |
| HR (95%CI) **^†^** | 1.0 (Reference) | 1.29 (0.98, 1.71) | 1.96 (0.99, 3.87) | | 0.027 | |
| Other cancer |  |  |  | |  | |
| No. of cases/Total | 174/10375 | 1852/75038 | 217/9320 | |  | |
| Incidence rate/10,000 Person-Years | 12.46 | 18.80 | 18.03 | |  | |
| HR (95%CI) **^†^** | 1.0 (Reference) | 1.41 (1.20, 1.66) | 1.34 (1.08, 1.65) | | 0.010 | |

Abbreviations: HR, hazard ratio; CI, confidence interval.

**^†^**Adjust for Age (as time scale), Gender (women, men), Education level (elementary school or below, middle school, high school or above), Occupation (coal miners, other blue collars, white-collar), Drinking (never, abstainer, current), Family history of cancer (No, Yes), and History of cardiovascular diseases (No, Yes).

**Table S7.** Life’s Essential 8 Trajectories with Risk of Subtypes Cancer

| Subtypes of Cancer | Life’s Essential 8 Trajectories | | |
| --- | --- | --- | --- |
|  | Stable-High | Stable-Moderate | Stable-Low |
| Lung cancer |  |  |  |
| No. of cases/Total | 153/19881 | 493/43718 | 180/13952 |
| Incidence rate/10,000 Person-Years | 7.94 | 11.85 | 13.71 |
| HR (95%CI) **^†^** | 1.0 (Reference) | 1.21 (1.01, 1.46) | 1.38 (1.10, 1.73) |
| Liver cancer |  |  |  |
| No. of cases/Total | 45/19881 | 134/43718 | 50/13952 |
| Incidence rate/10,000 Person-Years | 2.33 | 3.21 | 3.80 |
| HR (95%CI) **^†^** | 1.0 (Reference) | 0.99 (0.70, 1.40) | 1.08 (0.71, 1.65) |
| Gastric cancer |  |  |  |
| No. of cases/Total | 43/19881 | 115/43718 | 44/13952 |
| Incidence rate/10,000 Person-Years | 2.23 | 2.76 | 3.34 |
| HR (95%CI) **^†^** | 1.0 (Reference) | 1.05 (0.73, 1.51) | 1.28 (0.82, 2.00) |
| Colorectal cancer |  |  |  |
| No. of cases/Total | 36/19881 | 134/43718 | 55/13952 |
| Incidence rate/10,000 Person-Years | 1.87 | 3.22 | 4.18 |
| HR (95%CI) **^†^** | 1.0 (Reference) | 1.60 (1.09, 2.34) | 2.06 (1.32, 3.24) |
| Breast cancer |  |  |  |
| No. of cases/Total | 107/19881 | 94/43718 | 13/13952 |
| Incidence rate/10,000 Person-Years | 5.56 | 2.26 | 0.99 |
| HR (95%CI) **^†^** | 1.0 (Reference) | 1.10 (0.83, 1.46) | 1.17 (0.65, 2.11) |
| Other cancer |  |  |  |
| No. of cases/Total | 288/19881 | 808/43718 | 245/13952 |
| Incidence rate/10,000 Person-Years | 15.00 | 19.49 | 18.72 |
| HR (95%CI) **^†^** | 1.0 (Reference) | 1.22 (1.06, 1.40) | 1.18 (0.98, 1.41) |

Abbreviations: HR, hazard ratio; CI, confidence interval.

**^†^** Adjust for Age (as time scale), Gender (women, men), Education level (elementary school or below, middle school, high school or above), Occupation (coal miners, other blue collars, white-collar), Drinking (never, abstainer, current), Family history of cancer (No, Yes), and History of cardiovascular diseases (No, Yes).

**Table S8.** Stratified Analyses of between Baseline Life’s Essential 8 Status and Cancer in Full Models**^†^**

| Subgroups | Life’s Essential 8 Status | | | *P*-trend | *P*-interaction |
| --- | --- | --- | --- | --- | --- |
|  | High | Moderate | Low |  |  |
| Age (years) |  |  |  |  | 0.042 |
| <45 | 1.0 (Reference) | 1.36 (1.09, 1.70) | 1.81 (1.27, 2.59) | 0.001 |  |
| 45-65 | 1.0 (Reference) | 1.14 (0.99, 1.30) | 1.17 (0.98, 1.39) | 0.106 |  |
| >65 | 1.0 (Reference) | 0.92 (0.75, 1.13) | 0.76 (0.57, 1.03) | <0.001 |  |
| Gender |  |  |  |  | 0.391 |
| Female | 1.0 (Reference) | 1.51 (1.28, 1.78) | 1.36 (0.86, 2.18) | <0.001 |  |
| Male | 1.0 (Reference) | 1.15 (1.01, 1.31) | 1.16 (0.99, 1.36) | 0.113 |  |
| Education level |  |  |  |  | 0.853 |
| Elementary school or below | 1.0 (Reference) | 1.03 (0.73, 1.46) | 0.97 (0.66, 1.42) | 0.639 |  |
| Middle school | 1.0 (Reference) | 1.17 (1.04, 1.32) | 1.17 (0.99, 1.38) | 0.061 |  |
| High school or above | 1.0 (Reference) | 1.78 (1.42, 2.23) | 2.03 (1.47, 2.83) | <0.001 |  |
| Occupation |  |  |  |  | 0.347 |
| Coalminers | 1.0 (Reference) | 1.18 (0.92, 1.52) | 1.21 (0.92, 1.60) | 0.304 |  |
| Other blue collars | 1.0 (Reference) | 1.24 (1.10, 1.39) | 1.25 (1.05, 1.49) | 0.003 |  |
| White collars | 1.0 (Reference) | 1.71 (1.24, 2.37) | 1.38 (0.84, 2.29) | 0.042 |  |
| Alcohol consumption |  |  |  |  | 0.926 |
| Never | 1.0 (Reference) | 1.30 (1.16, 1.45) | 1.39 (1.14, 1.69) | <0.001 |  |
| Abstainer | 1.0 (Reference) | 1.18 (0.57, 2.43) | 1.13 (0.52, 2.46) | 0.999 |  |
| Current | 1.0 (Reference) | 1.17 (0.92, 1.50) | 1.14 (0.88, 1.48) | 0.779 |  |
| Family history of cancer |  |  |  |  | 0.108 |
| No | 1.0 (Reference) | 1.24 (1.12, 1.37) | 1.25 (1.09, 1.43) | 0.002 |  |
| Yes | 1.0 (Reference) | 3.81 (1.17, 12.40) | 3.80 (1.06, 13.66) | 0.127 |  |
| History of cardiovascular diseases |  |  |  |  | 0.908 |
| No | 1.0 (Reference) | 1.26 (1.14, 1.40) | 1.28 (1.11, 1.46) | 0.001 |  |
| Yes | 1.0 (Reference) | 1.01 (0.47, 2.16) | 0.98 (0.44, 2.21) | 0.912 |  |

**^†^**Adjust for Age (as time scale), Gender (women, men), Education level (elementary school or below, middle school, high school or above), Occupation (coal miners, other blue collars, white-collar), Drinking (never, abstainer, current), Family history of cancer (No, Yes), and History of cardiovascular diseases (No, Yes). The strata option has been implemented to tackle the variable violated the proportional assumption. Of note, the examined variable did not adjust.

**Table S9.** Stratified Analyses of between Life’s Essential 8 Trajectories and Cancer in Full Models**^†^**

| Subgroups | Life’s Essential 8 Trajectories | | | *P*-interaction |
| --- | --- | --- | --- | --- |
|  | Stable-Low | Stable-Middle | Stable-High |  |
| Age (years) |  |  |  | 0.303 |
| <45 | 1.0 (Reference) | 1.09 (0.76, 1.55) | 1.52 (0.91, 2.54) |  |
| 45-65 | 1.0 (Reference) | 1.18 (1.05, 1.33) | 1.29 (1.12, 1.50) |  |
| >65 | 1.0 (Reference) | 0.98 (0.83, 1.16) | 0.95 (0.76, 1.21) |  |
| Gender |  |  |  | 0.139 |
| Female | 1.0 (Reference) | 1.43 (1.21, 1.69) | 1.35 (0.95, 1.91) |  |
| Male | 1.0 (Reference) | 1.11 (0.99, 1.24) | 1.21 (1.06, 1.38) |  |
| Education level |  |  |  | 0.540 |
| Elementary school or below | 1.0 (Reference) | 1.09 (0.81, 1.47) | 0.94 (0.67, 1.34) |  |
| Middle school | 1.0 (Reference) | 1.18 (1.05, 1.31) | 1.21 (1.05, 1.39) |  |
| High school or above | 1.0 (Reference) | 1.30 (1.04, 1.61) | 1.96 (1.49, 2.58) |  |
| Occupation |  |  |  | 0.282 |
| Coalminers | 1.0 (Reference) | 1.11 (0.90, 1.37) | 1.26 (1.01, 1.59) |  |
| Other blue collars | 1.0 (Reference) | 1.21 (1.09, 1.36) | 1.22 (1.05, 1.42) |  |
| White collars | 1.0 (Reference) | 1.21 (0.90, 1.63) | 1.46 (0.98, 2.18) |  |
| Alcohol consumption |  |  |  | 0.232 |
| Never | 1.0 (Reference) | 1.21 (1.09, 1.35) | 1.25 (1.06, 1.48) |  |
| Abstainer | 1.0 (Reference) | 1.02 (0.62, 1.66) | 1.00 (0.57, 1.73) |  |
| Current | 1.0 (Reference) | 1.17 (0.97, 1.42) | 1.29 (1.05, 1.59) |  |
| Family history of cancer |  |  |  | 0.273 |
| No | 1.0 (Reference) | 1.18 (1.07, 1.29) | 1.26 (1.12, 1.42) |  |
| Yes | 1.0 (Reference) | 1.62 (0.78, 3.36) | 1.59 (0.70, 3.62) |  |
| History of cardiovascular diseases |  |  |  | 0.759 |
| No | 1.0 (Reference) | 1.20 (1.09, 1.32) | 1.27 (1.13, 1.43) |  |
| Yes | 1.0 (Reference) | 0.71 (0.40, 1.26) | 0.95 (0.51, 1.75) |  |

**^†^**Adjust for Age (as time scale), Gender (women, men), Education level (elementary school or below, middle school, high school or above), Occupation (coal miners, other blue collars, white-collar), Drinking (never, abstainer, current), Family history of cancer (No, Yes), and History of cardiovascular diseases (No, Yes). The strata option has been implemented to tackle the variable violated the proportional assumption. Of note, the examined variable did not adjust.

**Table S10.** Hazard Ratios of Life’s Essential 8 with Risk of Cancer Using Fine-Gray Models

| Exposure | No. of cases/ Total | Incidence rate per 10,000 Person-Years | SHR (95% CI) | | |
| --- | --- | --- | --- | --- | --- |
|  |  |  | Model I^†‡^ | Model II^†§^ | Model III^†¶^ |
| Baseline LE8 |  |  |  |  |  |
| High | 435/10375 | 31.38 | 1.0 (Reference) | 1.0 (Reference) | 1.0 (Reference) |
| Moderate | 4114/75038 | 42.09 | 1.30 (1.17, 1.44) | 1.25 (1.13, 1.38) | 1.25 (1.13, 1.38) |
| Low | 511/9320 | 42.76 | 1.29 (1.13, 1.47) | 1.24 (1.08, 1.41) | 1.24 (1.08, 1.41) |
| *P-*trend |  |  | <0.001 | <0.001 | <0.001 |
| LE8 trajectories |  |  |  |  |  |
| Stable-High | 672/19881 | 35.19 | 1.0 (Reference) | 1.0 (Reference) | 1.0 (Reference) |
| Stable-Moderate | 1778/43718 | 43.14 | 1.21 (1.10, 1.33) | 1.18 (1.07, 1.30) | 1.18 (1.07, 1.29) |
| Stable-Low | 587/13952 | 45.10 | 1.25 (1.11, 1.40) | 1.25 (1.11, 1.41) | 1.25 (1.11, 1.40) |

Abbreviations: LE8, Life’s Essential 8; SHR, sub-distribution hazard ratio; CI, confidence interval; SHR, sub-distribution hazard ratio.

**^†^**The strata option has been implemented to tackle the variable violated the proportional assumption.
**^‡^**Adjust for Age (as time scale) and Gender (women, men).

**^§^**Adjust for Age (as time scale), Gender (women, men), Education level (elementary school or below, middle school, high school or above), and Occupation (coal miners, other blue collars, white-collar)

**^¶^**Adjust for Age (as time scale), Gender (women, men), Education level (elementary school or below, middle school, high school or above), Occupation (coal miners, other blue collars, white-collar), Drinking (never, abstainer, current), Family history of cancer (No, Yes), and History of cardiovascular diseases (No, Yes).

**Table S11.** The E-value for Life’s Essential 8 and Risk of Cancer or All-cause Death

| Exposure | HR (95% CI) | E-value | |
| --- | --- | --- | --- |
| Baseline Life’s Essential 8 Status | | | |
| Cancer |  |  |  |
| High | 1.0 (Reference) |  |  |
| Moderate |  |  |  |
| Low |  |  |  |
| All-cause death |  |  |  |
| High |  |  |  |
| Moderate |  |  |  |
| Low |  |  |  |
| Life’s Essential 8 Trajectories | | | |
| Cancer |  |  |  |
| Stable-High | 1.0 (Reference) |  |  |
| Stable-Moderate |  |  |  |
| Stable-Low |  |  |  |
| All-cause death |  |  |  |
| Stable-High |  |  |  |
| Stable-Moderate |  |  |  |
| Stable-Low |  |  |  |

**Table S12.** Hazard Ratios of Each Component of Life’s Essential 8 with Risk of All-cause Death Stratified by Sex

| Metrics | HR (95% CI) ^†^ for each 10-point decrease | | |
| --- | --- | --- | --- |
|  | Model for all | Model for women | Model for men |
| Diet | 0.98 (0.97, 0.99) | 0.96 (0.92, 1.00) | 0.98 (0.97, 0.99) |
| Physical activity | 0.95 (0.95, 0.96) | 0.93 (0.90, 0.95) | 0.95 (0.95, 0.96) |
| Smoking | 0.99 (0.99, 1.00) | 1.13 (1.10, 1.16) | 0.99 (0.99, 1.00) |
| Sleep health | 1.04 (1.03, 1.05) | 1.10 (1.08, 1.13) | 1.03 (1.02, 1.04) |
| BMI | 0.98 (0.98, 0.99) | 1.08 (1.05, 1.10) | 0.98 (0.97, 0.98) |
| Blood lipid | 1.00 (0.99, 1.01) | 1.07 (1.05, 1.09) | 0.99 (0.99, 1.00) |
| Blood glucose | 1.08 (1.07, 1.09) | 1.20 (1.18, 1.22) | 1.07 (1.06, 1.08) |
| Blood pressure | 1.11 (1.10, 1.11) | 1.17 (1.15, 1.20) | 1.10 (1.09, 1.11) |

Abbreviations: BMI, body mass index; LE8, Life’s Essential 8; HR, hazard ratio; CI, confidence interval.
**^†^** Adjust for Age (as time scale), Gender (women, men), Education level (elementary school or below, middle school, high school or above), Occupation (coal miners, other blue collars, white-collar), Drinking (never, abstainer, current), Family history of cancer (No, Yes), and History of cardiovascular diseases (No, Yes), and History of cardiovascular diseases (No, Yes). Of note, gender did not adjust when stratified by sex.

**Table S13.** Hazard Ratios of Each Component of Life’s Essential 8 with Risk of Cancer Stratified by Sex

| Metrics | HR (95% CI) ^†^ for each 10-point decrease | | |
| --- | --- | --- | --- |
|  | Model for all | Model for women | Model for men |
| Diet | 0.99 (0.97, 1.01) | 0.97 (0.93, 1.02) | 0.99 (0.97, 1.01) |
| Physical activity | 0.96 (0.95, 0.97) | 0.96 (0.93, 0.99) | 0.96 (0.94, 0.97) |
| Smoking | 1.03 (1.02, 1.03) | 1.01 (0.97, 1.06) | 1.03 (1.02, 1.03) |
| Sleep health | 1.02 (1.01, 1.03) | 1.05 (1.02, 1.08) | 1.01 (1.00, 1.02) |
| BMI | 0.99 (0.98, 0.99) | 1.06 (1.03, 1.08) | 0.97 (0.96, 0.98) |
| Blood lipid | 1.00 (0.99, 1.01) | 1.03 (1.01, 1.05) | 0.99 (0.98, 0.99) |
| Blood glucose | 1.02 (1.01, 1.03) | 1.04 (1.01, 1.06) | 1.01 (1.01, 1.03) |
| Blood pressure | 1.04 (1.03, 1.04) | 1.05 (1.03, 1.07) | 1.03 (1.03, 1.04) |

Abbreviations: BMI, body mass index; LE8, Life’s Essential 8; HR, hazard ratio; CI, confidence interval.
**^†^** Adjust for Age (as time scale), Gender (women, men), Education level (elementary school or below, middle school, high school or above), Occupation (coal miners, other blue collars, white-collar), Drinking (never, abstainer, current), Family history of cancer (No, Yes), and History of cardiovascular diseases (No, Yes), and History of cardiovascular diseases (No, Yes). Of note, gender did not adjust when stratified by sex.

**Table S14.** Hazard Ratios of Life’s Essential 8 with Risk of All-cause Death after Extending the Period of Trajectory Construction to Six Years

| Exposure | No. of cases/ Total | Incidence rate per 1000 Person-Years | HR (95% CI) | | |
| --- | --- | --- | --- | --- | --- |
|  |  |  | Model 1^†^ | Model 2^‡^ | Model 3^§^ |
| LE8 Trajectories |  |  |  |  |  |
| High-Decreasing | 281/8172 | 4.51 | 1.0 (Reference) | 1.0 (Reference) | 1.0 (Reference) |
| Moderate-Decreasing | 1364/23149 | 7.80 | 1.40 (1.23, 1.60) | 1.33 (1.17, 1.51) | 1.32 (1.16, 1.50) |
| Moderate-Stable | 1536/19274 | 10.62 | 1.78 (1.56, 2.03) | 1.68 (1.47, 1.91) | 1.71 (1.50, 1.95) |
| Low-Stable | 305/3861 | 10.49 | 1.70 (1.44, 2.01) | 1.62 (1.37, 1.91) | 1.70 (1.44, 2.02) |

Abbreviations: LE8, Life’s Essential 8; HR, hazard ratio; CI, confidence interval.

**^†^**Adjust for Age (as time scale) and Gender (women, men).

**^‡^**Adjust for Age (as time scale), Gender (women, men), Education level (elementary school 6 or below, middle school, high school or above), and Occupation (coal miners, other blue collars, white-collar)

**^§^**Adjust for Age (as time scale), Gender (women, men), Education level (elementary school or below, middle school, high school or above), Occupation (coal miners, other blue collars, white-collar), Drinking (never, abstainer, current), Family history of cancer (No, Yes), and History of cardiovascular diseases (No, Yes).

**Table S15.** Hazard Ratios of Life’s Essential 8 with Risk of Cancer after Extending the Period of Trajectory Construction to Six Years

| Exposure | No. of cases/ Total | Incidence rate per 10,000 Person-Years | HR (95% CI) | | |
| --- | --- | --- | --- | --- | --- |
|  |  |  | Model 1^†^ | Model 2^‡^ | Model 3^§^ |
| Trajectory of LE8 |  |  |  |  |  |
| High-Decreasing | 217/8172 | 35.19 | 1.0 (Reference) | 1.0 (Reference) | 1.0 (Reference) |
| Moderate-Decreasing | 690/23149 | 39.84 | 1.15 (0.98, 1.35) | 1.11 (0.95, 1.30) | 1.11 (0.95, 1.30) |
| Moderate-Stable | 654/19274 | 45.74 | 1.33 (1.13, 1.56) | 1.29 (1.10, 1.52) | 1.30 (1.10, 1.53) |
| Low-Stable | 143/3861 | 49.69 | 1.45 (1.16, 1.81) | 1.45 (1.16, 1.81) | 1.46 (1.17, 1.83) |

Abbreviations: LE8, Life’s Essential 8; HR, hazard ratio; CI, confidence interval.

**^†^**Adjust for Age (as time scale) and Gender (women, men).

**^‡^**Adjust for Age (as time scale), Gender (women, men), Education level (elementary school 6 or below, middle school, high school or above), and Occupation (coal miners, other blue collars, white-collar)

**^§^**Adjust for Age (as time scale), Gender (women, men), Education level (elementary school or below, middle school, high school or above), Occupation (coal miners, other blue collars, white-collar), Drinking (never, abstainer, current), Family history of cancer (No, Yes), and History of cardiovascular diseases (No, Yes).


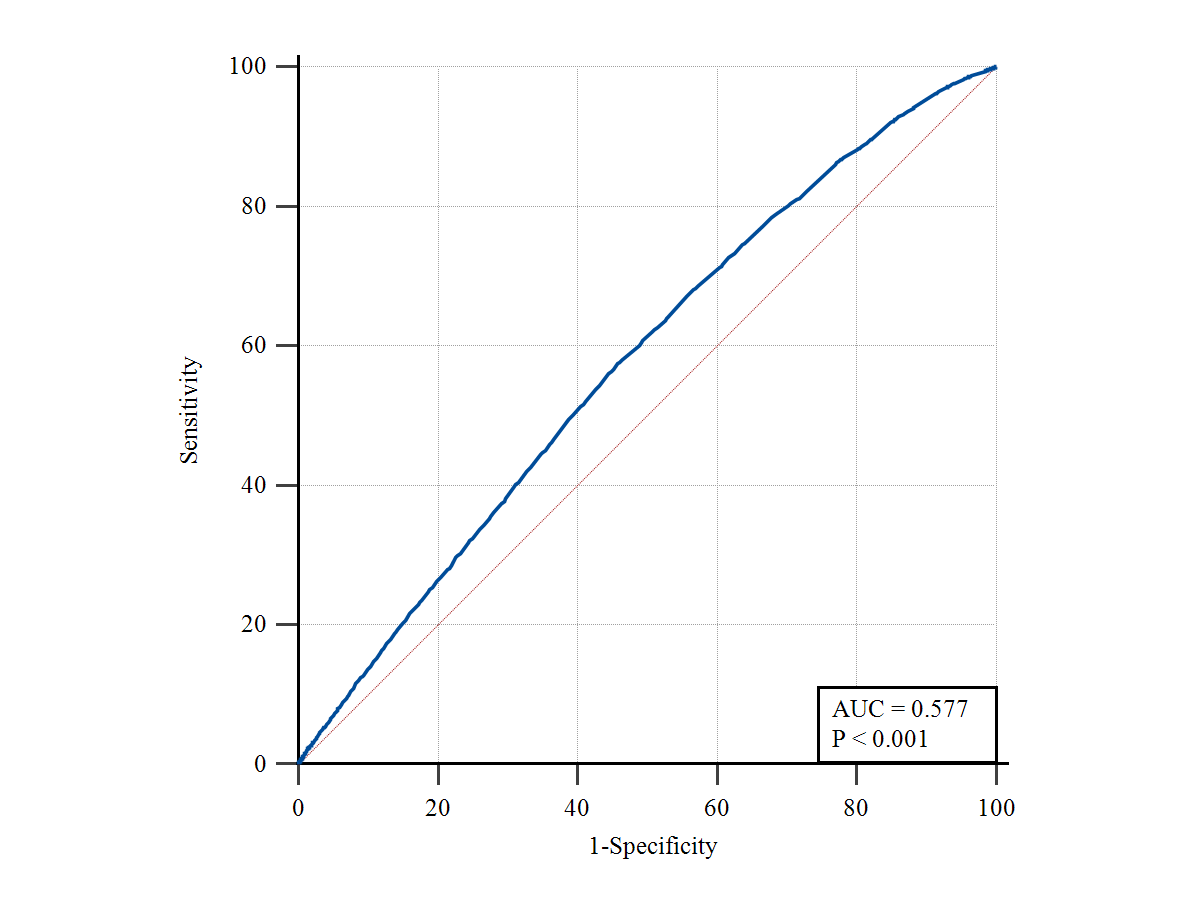
**
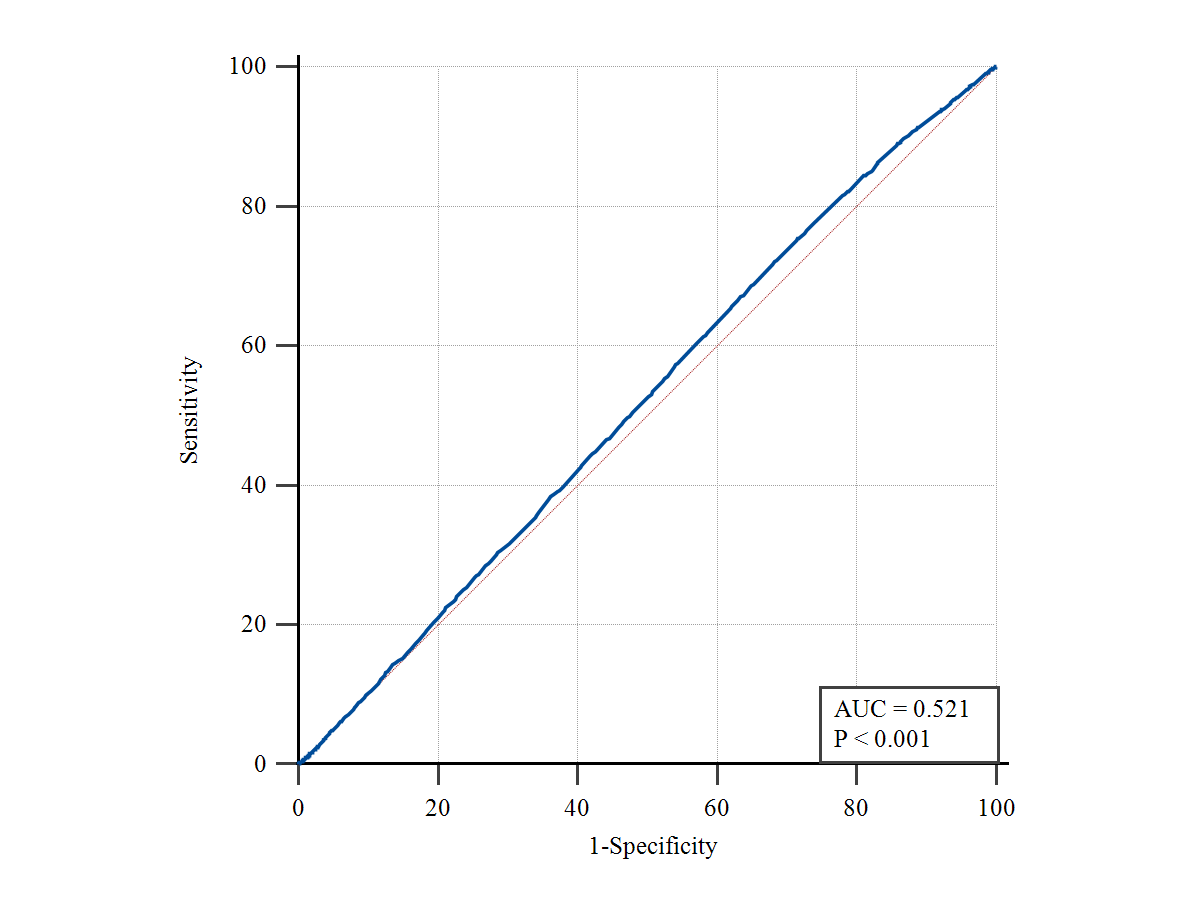
**

**Figure S1.** ROC Curves for the Association of Life’s Essential 8 with all-cause Death (right) and Incident Cancer (left)

**
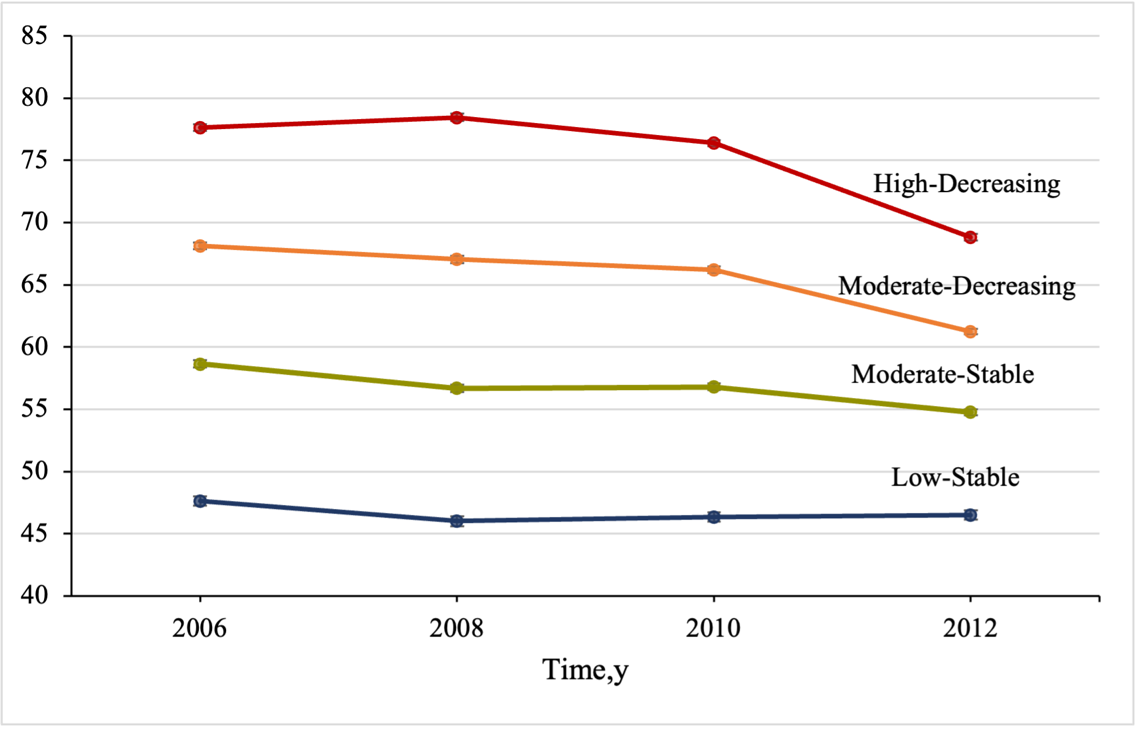
**

**Figure S2.** Mean Life’s Essential 8 Score in 2006, 2008, 2010, and 2012, According to 4 Life’s Essential 8 Score Trajectory Patterns

The Life’s Essential 8 Score range from 0 to 100, with the highest score representing the best health level. Error bars indicate 95% CI.
